# Association of Plasma Myeloperoxidase Level with Risk of Coronary Artery Disease in Patients with Type 2 Diabetes

**DOI:** 10.1155/2015/761939

**Published:** 2015-09-16

**Authors:** Ping Song, Jin Xu, Yongfeng Song, Shiliang Jiang, Haitao Yuan, Xu Zhang

**Affiliations:** ^1^Department of Endocrinology, Shandong Provincial Hospital, Shandong University, No. 324, Jing 5 Road, Jinan, Shandong 250021, China; ^2^Shandong Clinical Medical Center of Endocrinology and Metabolism, No. 324, Jing 5 Road, Jinan, Shandong 250021, China; ^3^Institute of Endocrinology and Metabolism, Shandong Academy of Clinical Medicine, No. 324, Jing 5 Road, Jinan, Shandong 250021, China; ^4^Department of Cardiology, Shandong Provincial Hospital, Shandong University, No. 324, Jing 5 Road, Jinan, Shandong 250021, China

## Abstract

*Aims*. This study aimed to investigate whether the change of plasma myeloperoxidase (MPO) level would be associated with the incidence of coronary artery disease (CAD) among diabetic patients. *Methods*. 339 patients with type 2 diabetes mellitus (DM) underwent coronary angiography. Of them, 204 cases had CAD and were assigned to CAD group and 135 cases without CAD were assigned to non-CAD group. *Results*. Compared to non-CAD group, CAD group had higher level of plasma MPO (*p* < 0.01). Multiple linear regression analysis showed that plasma MPO level was correlated with Gensini score. Multiple logistic analysis showed that the odds ratios for CAD across increasing tertiles of MPO level were 1.191 (0.971–1.547) and 1.488 (1.115–2.228) (*p* = 0.048, *p* = 0.009 versus 1st tertile of MPO level, resp.) by adjusting for age, sex, and other conventional risk factors for CAD. The subjects were stratified into nine groups according to tertiles of MPO and HbA1c. The odds ratio for CAD was significantly higher in group with highest levels of MPO and HbA1c (OR = 4.08, *p* < 0.01). *Conclusion*. Plasma MPO level was positively correlated with the degree of coronary artery stenosis in type 2 diabetic patients, and increasing blood glucose might amplify the association between MPO and CAD.

## 1. Introduction

The incidence of cardiovascular disease among diabetic patients is 2–4 times higher than that in nondiabetic population. Cardiovascular event is also the leading cause of mortality for diabetic patients [1]. To prevent the occurrence of cardiovascular events is important to reduce mortality in diabetic patients. Inflammation caused by oxidative stress is the primary mechanism of the formation of atherosclerosis [2]. Myeloperoxidase (MPO) is involved in the process of oxidative stress and plays a pathophysiological role in atherogenesis [3]. Clinical studies have shown that elevated blood MPO level was closely related to higher risk of coronary artery disease (CAD) [4]. Thus it could serve as a marker for the incidence of cardiovascular events [4–6]. Even in healthy people, high MPO level was considered as a risk factor for CAD and could predict future cardiovascular events [7]. In patients with type 2 diabetes, the risk of diabetic macrovascular complication was associated with hyperglycemia. Each 1% reduction in mean HbA(1c) was associated with reductions in risk of 14% for myocardial infarction [8], and 1% increase in HbA1c level among patients with type 2 diabetes was 15% increase for coronary heart disease [9]. The MPO level was higher in diabetic patients than that in nondiabetic population [10–12], and increasing MPO levels were associated with greater progression of atherosclerosis in diabetic patients [13], but little is known about the association between MPO level and the presendence and severity of CAD in subjects with type 2 diabetes. High glucose stimulates the production of hydrogen peroxide (H_2_O_2_), and MPO can use H_2_O_2_ as physiological substrate to form hypochlorous acid, so high glucose results in increasing MPO activity [14]. We speculated that the relationship between MPO and CAD may be stronger on a background of hyperglycemia.

Therefore, in this study, we determined the relationship between MPO levels and the incidence of CAD in patients with type 2 diabetes. Furthermore, to produce unbiased estimate of correlation, data were adjusted for other cardiovascular risk factors.

## 2. Subjects, Materials, and Methods

### 2.1. Subject Population

Based on WHO diagnostic criteria for type 2 diabetes [15], we enrolled 382 patients with type 2 diabetes mellitus (DM) who were consecutively referred and underwent their first coronary angiography because of suspected coronary atherosclerosis at our hospital between April 2012 and October 2014. Those with medical illnesses such as unstable CAD, acute coronary syndrome, history of myocardial infarction, heart failure, infectious or inflammatory disease, chronic hepatic and renal dysfunction (including serum alanine aminotransferase > 120 IU/L, aspartate aminotransaminase > 80 IU/L, and serum creatinine > 2.0 mg/dL), and history of cerebral infarction were excluded. Thus, the present analysis includes 339 patients. The patients were divided into 2 groups based on the results of coronary angiography: (1) CAD group: DM patients with CAD, *n* = 204; (2) non-CAD group: DM patients without CAD, *n* = 135. All subjects were of Chinese Han ethnicity and had no significant differences in geographic location and income. Two-milliliter fasting blood was taken from each subject in early morning, mixed with EDTA, and used for the determination of plasma MPO concentration. The study was approved by the Medical Ethics Committee of the Hospital, and all study subjects signed informed consent.

### 2.2. Clinical and Biochemical Measurements

#### 2.2.1. Measurement of Plasma MPO Concentration

The test was performed using ELISA kit (R&D, USA) according to manufacturer's instruction.

#### 2.2.2. Glucose and Lipids in the Blood Were Measured according to Professional Guideline

The level of glycosylated hemoglobin (HbA1c) was measured with ADAMSTMA1c HA-8160 automated glycated hemoglobin analyzer (Arkray, Japan) and high performance liquid chromatography (HPLC).

### 2.3. Coronary Angiography and Determination of Coronary Artery Stenosis

CAD was defined as coronary artery stenosis ≥50%. The degree of stenosis was evaluated using Gensini scoring system [16]. The stenosis of each coronary artery was scored as 0 if there were no abnormalities, 1 if stenosis was ≤ 25%, 2 if stenosis was between 26% and 50%, 4 if stenosis was between 51% and 75%, 8 if stenosis was between 76% and 90%, 16 if stenosis was between 91% and 99%, and 32 if there was 100% occlusion. The score of each coronary artery was then calculated by multiplying the stenosis score with the coefficient given according to the location of lesions. The degree of coronary lesions for each patient was the total score of all coronary arteries.

### 2.4. Statistical Analysis

Data were analyzed by SPSS (16.0). Quantitative data was expressed as mean ± SD and categorical data was given as percentages. Between-groups differences were tested by Student's *t*-test or Mann-Whitney *U* test according to the data distribution, with or without normality. *χ* ^2^ test was used to compare categorical variables between the groups. For multiple groups analyses, analysis of variance and Kruskal-Wallis test were used to determine significant differences. Spearman correlation coefficients between MPO level and other indicators were calculated. Multiple linear regression model was used for Gensini score and MPO level. Multiple logistic regression analysis was also performed among diabetic patients to identify factors (including age, duration of disease, and MPO level) that are associated with the occurrence of CAD. Statistical significance was defined at *p* < 0.05.

### 2.5. Results

**Figure 1:**
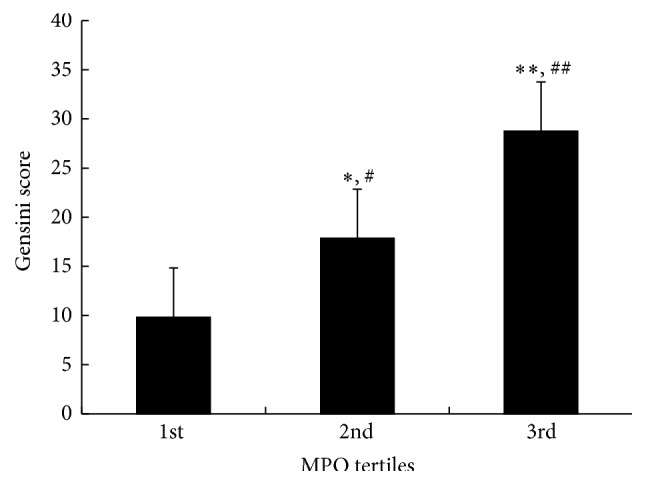
The comparison of Gensini score with tertiles of MPO. MPO: myeloperoxidase. ^*∗*^ *p* < 0.05, ^*∗∗*^ *p* < 0.01 versus 1st tertile of MPO. ^#^ *p* < 0.05, ^##^ *p* < 0.01 versus 2nd tertile of MPO.

(1) Clinical characteristics about patients' age, gender, blood pressure, and so forth were summarized in Table 1. The mean value of MPO in CAD group was significantly higher than that in non-CAD group (*p* < 0.01). There were no significant differences between the CAD group and non-CAD group with respect to age, BMI, and DBP.

(2) The diabetic patients were divided into 3 groups based on the tertile of MPO level and Gensini scores were compared among subgroups. As shown in Figure 1, the mean value of Gensini score showed significant increasing tendency according to tertiles of MPO (*p* < 0.01).

(3) Correlation and regression analysis: among diabetic patients, Spearman correlation analysis showed that plasma MPO level was positively correlated with systolic blood pressure (SBP, *r* = 0.195, *p* = 0.012) and negatively correlated with high-density lipoprotein cholesterol (HDL-C, *r* = −0.265, *p* < 0.001). Multiple linear regression analysis was performed with Gensini score as the dependent variable and MPO as independent variables, according to tertiles of HbA1c. Plasma MPO levels were significantly associated with Gensini scores and the de beta coefficient was gradually increased with rise of HbA1c, even after adjustment for age, sex, and other risk factors of CAD (the 1st HbA1c tertile group: *β* = 0.154, *p* = 0.038; the 2nd HbA1c tertile group: *β* = 0.185, *p* = 0.025; the 3rd HbA1c tertile group: *β* = 0.216, *p* = 0.008, resp.), as shown in Table 2.

Multiple logistic regression analysis showed that MPO level was significantly associated with the occurrence of CAD among diabetic patients (OR = 1.275, 95% CI 1.067–1.526, *p* = 0.018), as shown in Table 3. The odds ratios (95% CI) for CAD according to the tertiles of plasma MPO level were showed in Table 4. Compared to 1st tertiles of plasma MPO level, the odds ratio for CAD in the 2nd tertiles of plasma MPO level was 1.19 (95% CI 0.971–1.547, *p* = 0.048) and the odds ratio for CAD in the 3rd tertiles of plasma MPO level was 1.488 (95% CI 1.115–2.228, *p* = 0.009) by adjusting for age, sex, and other conventional risk factors for CAD.

**Figure 2:**
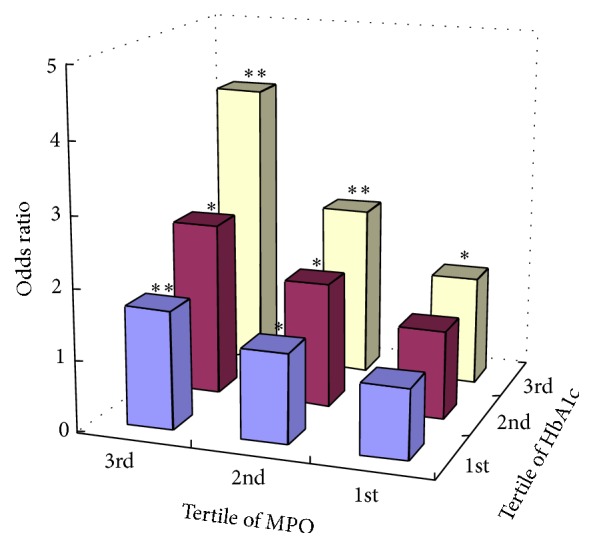
Odds ratio for CAD associated with tertiles of plasma MPO level and tertiles of HbA1c level compared to the group with 1st MPO tertile and 1st HbA1c tertile. ^*∗*^ *p* < 0.05, ^*∗∗*^ *p* < 0.01.

To investigate whether blood glucose exhibited synergistic elevation in risk for CAD with MPO, the study participants were stratified into nine groups according to MPO tertiles and tertiles of HbA1c. As shown in Figure 2, comparing to the odds ratio for CAD in the group with 1st MPO tertile and 1st HbA1c tertile, the odds ratio for CAD tended to be more significant among group with 3rd MPO level and 3rd HbA1c level (OR = 4.08, 95% CI 2.35–6.34, *p* < 0.01).

## 3. Discussion

In this study, plasma MPO concentration was demonstrated to be significantly higher in diabetic patients with the complication of CAD than that in the non-CAD group. The Gensini score, which was served to assess the degree of coronary stenosis, increased progressively along with the increasing plasma MPO level. Multiple linear regression analysis showed that this relationship between MPO and Gensini score in strata of HbA1c was stronger in individuals with the high HbA1c levels. Multiple logistic regression analysis revealed that plasma MPO level was an influential factor for CAD among diabetic patients. The odds ratio for risk of CAD was significantly increased in the highest MPO tertile and HbA1c combined with MPO strengthened the odds ratio for risk of CAD.

Diabetic patients are prone to atherosclerosis, and the role of inflammatory factors in the development of atherosclerosis remains to be an interesting topic [2]. MPO is a heme protein produced primarily by neutrophils. It catalyzes the production of hypochlorous acid, tyrosyl radical, and reactive nitrogen free radicals, which can then promote the occurrence of vascular inflammation. Studies have suggested that MPO, through the modification of LDL, could promote the formation of foam cell and the formation and rupture of the plaque and therefore deserved great attention in understanding the development of coronary artery disease [17]. Düzgünçinar et al. [18] found that blood MPO level was correlated with the Gensini score in patients with CAD. The results of current study from diabetic patients were consistent with these reports. The increase of plasma MPO level was associated with both the occurrence of CAD and the severity of coronary artery stenosis in diabetic patients.

Hyperglycemia can stimulate the production of hydrogen peroxide, resulting in an increase of MPO activity. Hyperglycemia also damages artery endothelial cells and accelerates the progression of atherosclerosis. HbA1c is a marker of long-term glycemic exposure, reflecting an average blood glucose level in the prior 2-3 months, and has less biological variability than fasting glucose. For such reasons, HbA1c was recommended for risk stratification analysis. In this study, the HbA1c level was not statistically significantly associated with plasma MPO level, but the association of MPO with the risk of CAD was exaggerated in high HbA1c level, indicating that high concentration of glucose could interact with MPO and subsequently enhanced the risk of CAD.

This study showed that SBP was associated with plasma MPO level. The increased release of inflammatory cytokines such as TNF-*α* and IL-8 during high blood pressure and the activation of renin-angiotensin system could lead to neutrophil activation and respiratory burst [19, 20], which might promote the release of MPO from neutrophils and resulted in the increase of MPO in the blood. The increase of blood MPO level can exacerbate the damage of vascular endothelial cells, reduce the elasticity of the blood vessel walls, and affect patients' blood pressure. In addition, a stronger association was found between MPO level and high blood pressure at high blood glucose concentration [21]. In this study, multiple logistic regression analysis showed that MPO, SBP, and HbA1c were all associated with the occurrence of coronary artery disease among diabetic patients, suggesting that the interactions among high blood pressure, blood glucose, and MPO accelerated the progress of atherosclerosis. Dyslipidemia is a major risk factor for atherosclerosis. Diabetic patients often have abnormal lipoprotein metabolism. HDL-C not only can reduce blood cholesterol level, but also has antioxidant and anti-inflammatory effects. Studies have reported that HDL-C can inhibit the neutrophil activation [22, 23]. This study showed that HDL-C was negatively correlated with plasma MPO concentration. The neutrophil activity and MPO release were increased with the reduction of HDL-C, resulting in an elevation of plasma MPO level.

One limitation of this study is that only plasma MPO concentration, not its activity, was measured. However, previous studies have already demonstrated a strong relationship between plasma MPO concentration and MPO activity [24]. It is plausible to speculate that the current higher plasma MPO concentration reflects the higher MPO activity. Given that MPO is activated in the patients' vascular wall and its impact on the arterial injury is not clear yet, it would be of interest to collect samples of coronary artery from diabetic patients in the future study to further investigate the mechanism of MPO-related coronary artery injury.

In conclusion, patients with type 2 diabetes had increased plasma MPO concentrations. Those complicated with CAD had even higher levels of plasma MPO level. Plasma MPO level and Gensini score were positively correlated, suggesting that MPO might be an influential factor in the progression of CAD among diabetics and might serve as a new target for the diagnosis and treatment of CAD in patients with type 2 diabetes.

## Figures and Tables

**Table 1:** Clinical characteristics of subjects in non-CAD and CAD groups.

	Non-CAD (*n* = 135)	CAD (*n* = 204)	*p* value
Age (y)	58.9 ± 10.3	60.2 ± 11.5	0.425
Sex (male %)	69.6%	74.0%	0.175
Disease duration (year)	6.3 ± 3.6	8.4 ± 3.5	0.028
BMI (kg/m^2^)	26.3 ± 4.5	26.7 ± 4.6	0.656
WHR	0.88 ± 0.10	0.97 ± 0.17	0.044
SBP (mmHg)	138.6 ± 14.5	147.1 ± 21.2	0.022
DBP (mmHg)	83.9 ± 8.2	85.3 ± 9.4	0.195
Current smoker, *n* (%)	27 (20%)	86 (42.2%)	0.018
TC (mmol/L)	5.25 ± 0.86	5.69 ± 1.13	0.014
TG (mmol/L)	2.24 ± 0.63	2.57 ± 0.62	0.036
HDL-C (mmol/L)	1.06 ± 0.15	0.95 ± 0.13	0.012
LDL-C (mmol/L)	3.38 ± 0.63	3.65 ± 0.92	0.042
FBG (mmol/L)	7.86 ± 1.65	9.23 ± 2.54	0.015
HbA1c (%)	7.28 ± 0.93	8.89 ± 1.38	0.006
MPO (ng/mL)	58.3 ± 14.7	78.5 ± 19.8	0.002
Insulin therapy (%)	21 (22.1%)	45 (21%)	0.558
Sulfonylurea (%)	43 (45.3%)	103 (48.1%)	0.476
Metformin (%)	68 (71.6%)	67 (78%)	0.522
Thiazolidinedione (%)	20 (21.1%)	38 (17.8%)	0.485
ARB or ACE inhibitors (%)	45 (47.4%)	128 (62.1%)	0.079
Calcium channel blocker (%)	24 (25.3%)	62 (29%)	0.245
Beta blocker (%)	14 (14.7%)	36 (16.8%)	0.317
Statins (%)	58 (61.1%)	146 (68.2%)	0.326

**Table 2:** Multivariable linear regression analysis for the relation between MPO and Gensini score in strata of HbA1c.

	1st tertile of HbA1c		2nd tertile of HbA1c		3rd tertile of HbA1c	
	(<7.2%)		(7.2%–8.5%)		(>8.5%)	
	*β*	*p* value	*β*	*p* value	*β*	*p* value
Model 1	0.165	0.027	0.198	0.012	0.238	0.002
Model 2	0.154	0.038	0.185	0.025	0.216	0.008

Model 1: adjusting for age, sex.

Model 2: adjusting for age, sex, BMI, smoking, SBP, DBP, LDL-C, HDL-C, and TG.

**Table 3:** Multiple logistic regression for the risk of CAD in diabetes patients.

Variable	OR	95% CI	*p* value
SBP	3.152	1.19–7.96	0.003
Age	2.082	1.28–3.16	0.003
LDL-C	1.652	1.08–1.56	0.007
MPO	1.275	1.06–1.56	0.018
HbA1c	1.151	1.06–1.56	0.029

**Table 4:** Multivariable logistic regression analysis for CAD in strata of MPO level.

	OR (95% CI)		
	1st tertile of MPO	2nd tertile of MPO	3rd tertile of MPO
Model 1	1.0	1.253 (0.982–1.642)	1.505 (1.054–2.385)
*p* values		0.038	0.003
Model 2	1.0	1.217 (0.975–1.589)	1.492 (1.075–2.252)
*p* values		0.042	0.006
Model 3	1.0	1.191 (0.971–1.547)	1.488 (1.115–2.228)
*p* values		0.048	0.009

Model 1: adjusting for age, sex.

Model 2: adjusting for age, sex, BMI, smoking, SBP, DBP, LDL-C, HDL-C, and TG.

Model 3: adjusting for age, sex, BMI, smoking, SBP, DBP, LDL-C, HDL-C, TG, FBG, and HbA1c.

## Abbreviations

DM: Diabetes mellitus
CAD: Coronary artery disease
BMI: Body mass index
WHR: Waist-hip ratio
SBP: Systolic blood pressure
DBP: Diastolic blood pressure
TC: Total cholesterol
TG: Triglyceride
HDL-C: High-density lipoprotein cholesterol
LDL-C: Low-density lipoprotein cholesterol
FBG: Fasting blood glucose
HbA1c: Glycosylated hemoglobin
MPO: Myeloperoxidase.
